# Overexpression of miR-145 in U87 cells reduces glioma cell malignant phenotype and promotes survival after *in vivo* implantation

**DOI:** 10.3892/ijo.2014.2807

**Published:** 2014-12-23

**Authors:** YONG LU, MICHAEL CHOPP, XUGUANG ZHENG, MARK KATAKOWSKI, DING WANG, ELISE FRASER, MONIQUE NGUYEN, FENG JIANG

**Affiliations:** 1 Department of Neurology, Henry Ford Hospital, Detroit, MI, USA; 2 Department of Hematology/Oncology, Henry Ford Hospital, Detroit, MI, USA; 3 Department of Physics, Oakland University, Rochester, MI, USA

**Keywords:** miR-145, ADAM17, EGFR, glioma, migration, invasion, *in vivo*

## Abstract

In the present study, we sought to elucidate the effect of miR-145 on glioma cell progression and its mechanisms of action. We examined the effects of miR-145 on proliferation and invasion of U87 glioma cells and on capillary tube formation. Our data show that restoration of miR-145 in U87 glioma cells significantly reduced their *in vitro* proliferation, invasion and angiogenesis. However, decreased miR-145 expression promoted U87 glioma cell proliferation, invasion and angiogenesis, and reduced-expression of miR-145 increased ADAM17 and EGFR expression in U87 cells. Overexpression of miR-145 reduced ADAM17 and EGFR expression. VEGF secretion and VEGF expression were decreased by increased miR-145 expression in U87 cells and were reversed by miR-145 down-regulation *in vitro*. Nude mice with intracerebral implantation of U87 overexpressing miR-145 cells exhibited significantly reduced tumor growth and promoted survival compared with control groups. Taken together, these results suggest a role for miR-145 as a tumor suppressor which inhibits glioma cell proliferation, invasion and angiogenesis *in vitro* and reduces glioma growth *in vivo*.

## Introduction

High-grade gliomas are malignant as they are highly vascularized and invasive, and characterized by high incidence of recurrence and poor prognosis (1,2). The prognosis for patients with malignant gliomas has not significantly changed in recent years (3). Glioma cells infiltrate into the normal brain adjacent to the tumor causing treatment failure from conventional therapy, including surgery, radiotherapy and chemotherapy (4). Thus, median survival time has changed little and is ~15 months (1). Brain tumors constitute the second most frequent cause of cancer deaths in patients <15 years of age, the third-cause for adult men, and the fourth cause in women aged 15–34 years (5). Malignant gliomas are highly fatal and often strike patients in their most productive years.

MiRNAs, a class of small, non-encoding RNAs that transcriptionally or post- transcriptionally modulate the expression of their target genes, and have been implicated as regulatory molecules in various cancers from lung, breast, colon, prostate, head and neck, including melanoma and glioma (6,7). MiRNAs may alter the functions of oncogenes and/or tumor suppressor genes, and therefore, impact the biological behavior of these cancers. miR-145 is a tumor-suppressive miRNA that has been recently implicated in the regulation of cell growth in tumor cells through targeting c-myc, EGFR, OCT4, and MUC1 (8–11). However, little has been reported on whether miR-145 is associated with glioma progression.

ADAM17 (a.k.a. TACE), is involved in the ectodomain shedding of multiple membrane-bound ligands and cytokines, it has been implicated in diverse biological processes, including growth and inflammation (12). Currently, ADAM17 is best known as the primary secretase responsible for releasing the soluble form of tumor necrosis factor-α (TNF-α) from the plasma membrane. Of particular interest, ADAM17 has recently been identified as the primary sheddase for multiple epidermal growth factor receptor (EGFR) pro-ligands (13). EGFR ligand-binding results in receptor self-dimerization, auto-phosphorylation and subsequent activation of downstream PI3K/AKT and Ras/MAPK/ERK signaling pathways (14–16). EGFR is the prototype of a family of receptors with tyrosine kinases that participate in the control of differentiation, proliferation, and cell survival, as well as in the development of tumors of epidermal origin (16–19). The expression of EGFR is frequently upregulated in human glioma, and its overexpression correlates with poor prognosis (19).

Recently, we have demonstrated that expression of miR-145 is significantly downregulated in glioma cells as compared with normal brain tissue (20). miR-145 directly targets ADAM17 and binds to 3′ UTR of ADAM17 mRNA, decreasing ADAM17 protein expression. Our present study demonstrates that overexpression of miR-145 inhibits glioma cell proliferation, invasion, angiogenesis *in vitro*, and decreases glioma cell growth in an animal model, possibly through the mechanism of miR-145-related decrease of ADAM17 and EGFR protein expressions via the Erk/p-Erk pathway in U87 cells.

## Materials and methods

### Cell line and cell culture

Human glioblastoma U87, and mouse brain endothelial cells were obtained from the American Type Culture Collection (Manassas, VA, USA). The cells were grown in Dulbecco’s modified Eagle’s medium (DMEM; Invitrogen, Carlsbad, CA, USA) supplemented with 10% fetal bovine serum, 50 U/ml penicillin, and 50 mg/ml streptomycin. The U87 cells were maintained in a humidified 37°C incubator with 5% CO_2_, fed every 3 days with complete medium, and subcultured when confluence was reached.

### Transfection of U87 cells with miR-145 and MiRzip-145

The cDNAs encoding miR-145 and cel-67 were purchased from Genscript (Piscataway, NJ, USA). MiRzip-145 was purchased from System Biosciences (Mountain View, CA, USA). Transfections were done with the use of Lipofectamine 2000 (Life Technologies, Grand Island, NY, USA) according to the manufacturer’s instructions. Briefly, 1.5×10^5^ U87 glioma cells were seeded onto 6-well plate and incubated with 4 μg cDNA as suggested by the manufacturer. After 48 h, cells were selected using Puromycin (Sigma-Aldrich, St. Louis, MO, USA).

### Modified cell lines

Modified cell lines used in this study were stable transfection U87 cells with miR-145 cDNA plasmid stable transfection (U87-miR-145), U87 glioma cells with negative control cel-67 plasmid (U87-cel-67), and U87 glioma cells with miRzip-145 (targeting miR-145) plasmid stable transfection (U87-zip-145). The stable transfection was completed per the instructions of the Lipofectamine 2000 kit, according to the manufacturer’s instructions.

### Growth curve

U87 cells (1×10^4^) were plated into each well of 24-well plates containing DMEM with FBS at a concentration of 10%. Every 24 h medium was removed, adherent cells were trypsinized, and the total cell number of each well was quantified with the use of a hematocytometer. Cell number was expressed as an average of counts from three wells per time point per group.

### Bromodeoxyuridine incorporation assay

Cell proliferation was measured using BrdU incorporation assay. Forty thousand cells in 1,000 μl complete medium per well were placed into 24-well plates. After 48-h incubation, the culture medium was removed and cells were rinsed with PBS followed by incubation with BrdU (25 μg/ml) for 2 h and fixed in 4% paraformaldehyde for 30 min at room temperature. Following fixation, cells were incubated with 50% formaldehyde in 2X SSC at 65°C for 30 min and with 2N HCl at 37°C for 10 min. After incubation with 0.1 M boric acid at room temperature for 3 min, cells were rinsed with PBS and blocked with 1% bovine serum albumin at room temperature for 1 h, followed by incubation with an anti-BrdU antibody overnight at 4°C. Cells were then incubated with a FITC-conjugated secondary antibody to visualize BrdU positive labeled cells. Before mounting with coverslips, cells were incubated with 10 μg/ml of DAPI (4′-6-diamidino-2-phenylindole, Invitrogen) for 10 min. Four fields of cells were counted randomly in each well under a fluorescent microscope at ×10 magnification. The proliferation rate was expressed as the percentage of BrdU positive labeled cells divided by DAPI labeled cells.

### In vitro invasion assay

Matrigel chambers (BD Biosciences) were used to determine the effect of stable expression of miR-145 on invasiveness, according to the manufacturer’s protocol. Cells (1×10^5^) with stable transfection of miR-145, or zip-145 were plated into 6-well plates and incubated for 48 h. Then, glioma cells were re-suspended in 500 μl of serum-free medium and added to the upper chamber, while the lower chamber was filled with 0.5 ml of complete medium that served as a chemo-attractant. Cells were then incubated for 24 h at 37°C. After removal of cells from the upper surface of the membrane, cells on the lower surface of the membrane were stained with CellTracker™ Green (Molecular Probes, Eugene, OR, USA) for 30 min and fixed in 4% formaldehyde. Nine fields of cells were counted randomly in each well under a fluorescent microscope at ×100 magnification. All the experiments were done in duplicate and results were expressed as mean ± SEM of three independent experiments.

### Capillary-like tube formation assay

Briefly, 0.1 ml growth factor reduced Matrigel (Becton-Dickinson) was added per well, and mouse brain endothelial cells (MBECs, 2×10^4^ cells) were added and incubated in: i) regular cell culture medium (DMEM) for control; ii) cell culture medium from U87 only cell culture; iii) cell culture medium from U87 miR-145 cell culture; iv) cell culture medium from U87 zip-145 for ≤3 h. All assays were performed in triplicates. For quantitative measurements of capillary tube formation, Matrigel wells were digitized under a 4× objective (Olympus) for measurement of total tube length of capillary tube formation using the MCID image analysis system (Imaging Research, St. Catharines, Canada) at 3 h. Tracks of endothelial cells organized into networks of cellular cords (tubes) were counted and averaged in randomly selected three microscopic fields. The total tube length was calculated by MCID software in each field. The tube formation index was expressed as tube length (mm) per field.

### Western blot analysis

Cultures were rinsed with PBS and proteins were extracted in 500 ml RIPA lysis buffer (50 mM Tris-HCl, 150 mM NaCl, 1% NP-40, 0.5% sodium deoxycholate, 1 mM EDTA, 0.1% SDS and 0.01% sodium azide, pH 7.4). Equal amounts of proteins, as determined by the BCA protocol (Pierce, Rockford, IL, USA), run on 10% Tris-glycine gels (Invitrogen) and then transferred to PVDF membranes (Bio-Rad, Hercules, CA, USA). The membranes were blocked with 0.1% I-Block (Applied Biosystems, Foster City, CA, USA) in PBS-T (0.3% Tween-20) at room temperature for 1 h, followed by incubation with primary antibodies against ADAM17 (Abcam, Cambridge, MA, USA), EGFR (Cell Signaling, Danvers, MA, USA), Erk, p-Erk and actin (Santa Cruz, Santa Cruz, CA, USA) at 4°C overnight. The membranes were washed with PBS-T (0.3% Tween-20) and incubated for 1 h at room temperature with horseradish peroxidase-conjugated secondary antibodies (Bio-Rad Laboratories). Following washing, the specific proteins were detected using a West Pico chemiluminescent protein detection kit (Pierce). The experiment was repeated in triplicate. The densities of the bands were analyzed using the Scion image software (Frederick, MD, USA).

### TaqMan real-time PCR analysis of miR-145

Extraction of total RNA was obtained using Qiagen miRNeasy Mini isolation kit (Qiagen, Valencia, CA, USA) in accordance with the manufacturer’s instructions. To quantify the expression level of the miR-145 in glioma cells with either stable transfection of miR-145 or zip-145, TaqMan real-time PCR of miR-145 expression was carried out using TaqMan assay kits according to the manufacturer’s protocol. The kits contain TaqMan probes to detect mature miRNA in a two-step RT-PCR analysis. CDNAs were synthesized from total RNA using miRNA-specific primers and the TaqMan miRNA reverse transcription (RT) kit, according to the manufacturer’s instructions. The 15-μl reactions were incubated on a PCR system for 30 min at 16°C, 30 min at 42°C, 5 min at 85°C, and then held at 4°C. All reverse transcriptions and no-template controls were run at the same time. PCR amplification was carried out using sequence-specific primers on the Applied Biosystems 7500 real-time PCR system (Applied Biosystems). The assay was carried out in a 96-well optical plate at 95°C for 10 min, followed by 40 cycles of 95°C for 15 sec and 60°C for 60 sec. The threshold cycle (CT) data and baselines were determined using auto settings. U6 small RNA (RNU6B) was also identified using the TaqMan RNU6B assay kit for normalizing the levels of miR-145. Each sample that included no template was analyzed in triplicate.

### Human glioma cell implantation in nude mice

Nude mice (Nu/Nu Athymic, Charles River Breeding, MA, USA) with body weight of 15–25 g were anesthetized with ketamine (80 mg/kg) and xylazine (10 mg/kg) administered intraperitoneally (i.p.). After fixing the mouse in a stereotaxic device, the scalp was retracted to expose the cranium, and a 3–4-mm incision was made directly down the midline. With the use of a drill, a 2-mm craniotomy was made on the right hemisphere anterior to the coronal suture. A 10-ml Hamilton syringe was positioned to inject tumor cells stereotactically into the right hemisphere - 3.5 mm depth, 1.5 mm to the right, and 1.0 mm anterior to the bregma. U87 cells (7.5×10^5^), U87-cel-67, U87-miR-15, or U87-zip-145 were injected intracerebrally in a 7.5-μl volume. The craniotomy was covered with a film of polyvinyl chloride glued to the surrounding intact bone and the incision was closed with 4–0 silk suture. Tumor volume was evaluated in 6-mm paraffin sections in the tumor region stained with H&E. During sacrifice, mice were perfused via the left ventricle with heparinized saline followed by 4% paraformaldehyde. Tumor-bearing brain was processed, placed in paraffin, and subsequently five equally spaced sections (6 mm thick) were obtained from each of the 1-mm thick blocks encompassing the tumor. The sections were stained with H&E for microscopic analysis.

### Statistical analysis

Data are presented as mean and standard error of the mean. Statistical significance was analyzed by one-way ANOVA using GraphPad Prism software (version 4.0). P-value smaller than 0.05 (P<0.05) was considered statistically significant.

## Results

### miR-145 expression level in human glioma cell lines

**Figure 1:**
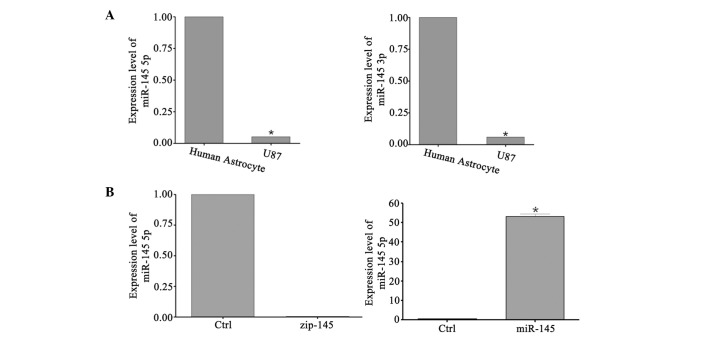
miR-145 expression levels in glioma cells. (A) Total RNA was isolated from normal human astroyctes and U87 gliomas. Real-time PCR was performed to analyze the expression ofmiR-145-3p and miR-145-5p. The relative expression of miR-145-3p and-5p was expressed as the ratio of the expression level of normal human astrocytes. ^*^P<0.05, as compared with norml human astrocytes. (B) U87 glioma cells were stably transfected with miR-145 and zip-145, and collected for real-time RT-PCR. Stable transfection of miR-145 significantly increased miR-145-5p in U87, as compared with non-transfected cells. However, compared to non-transfected U87 cells, stable transfection of zip-145 significantly decreased miR-145-5p. ^*^P<0.05 versus U87 glioma cells without transfection of miR-145 or zip-145.

Using real-time RT-PCR, we compared the expression levels of miR-145-3p and miR-145-5p in glioma cell lines with total RNA from normal human astrocytes. Human astrocytes were obtained from ScienCell Research Laboratories. As shown in Fig. 1, miR-145-5p and miR-145-3p expression levels were significantly lower (4.2 and 6.3%) in U87 compared to total RNA from human astrocytes. miR-145-5p directly targets ADAM17 mRNA and inhibits glioma cell proliferation and invasion (20). These data suggest the potential therapeutic value of miR-145 in malignant human gliomas.

### Establishment of U87 with stable expression of miR-145 and its inhibitor zip-145

We transfected U87 glioma cells with plasmids encoding miR-145 or zip-145, then added puromycin for high-expression clones. To determine miR-145 expression level with stable transfection of miR-145 and its inhibitor zip-145, Taqman real-time PCR analysis of miR-145 level was used to measure and confirm miR-145, zip-145 versus control U87 cells. As shown in Fig. 1, miR-145 expression level was significantly elevated (53.8-fold) in U87-145 cells compared with levels in control U87 cells. As for miR-145 overexpression in U87-zip-145 glioma cells, Fig. 1 shows that miR-145 expression level was significantly decreased to 0.4% compared to control U87 cells. The data suggested that stable transfection of miR-145 and zip-145 was established in the U87 cell lines.

### miR-145 decreases cell growth in U87 glioma cells

**Figure 2:**
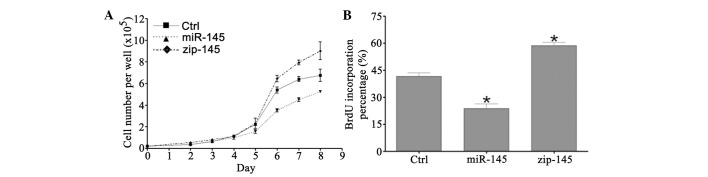
miR-145 inhibits glioma cell growth and proliferation *in vitro*. Overexpression decreased glioma proliferation. Downregulation of miR-145 by zip-145 increased glioma cell proliferation. (A) Growth curve; (B) BrdU incorporation assay for 48 h. ^*^P<0.05 versus control.

U87 cells transfected with miR-145 plasmid and control U87 cells were harvested and seeded at a density of 1×10^4^ cells/well in 24-well plates. The total cell number was quantified daily with a hematocytometer. Our data showed that overexpression of miR-145 significantly decreased the growth rate of U87 cells after 5 days culture (Fig. 2). Downregulation of miR-145 significantly increased the growth rate of U87 cells from 13.2 to 20.3% compared to control. We employed an additional method, the BrdU incorporation assay, to further test miR-145-mediated growth inhibition of glioma cells using miR-145 plasmid transfected U87 clones (Fig. 2). After 48 h of culture, miR-145 cells showed a 47.7% decrease in BrdU incorporation as compared with control. At the same time, we tested the downregulation of miR-145 on glioma cell proliferation. As compared with control U87 cells, miR-145 downregulation evidently increased BrdU incorporation after 48 h of culture by 145.4% as compared with the control group.

### Overexpression of miR-145 inhibits glioma cell invasiveness

**Figure 3:**
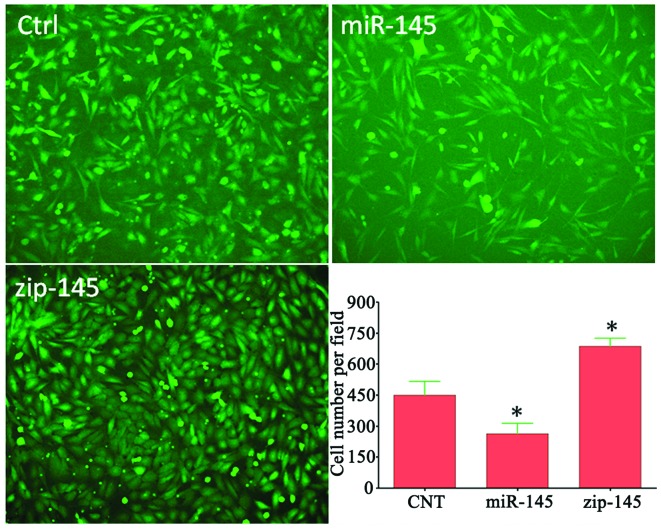
Effect of miR-145 expression on glioma cell invasiveness *in vitro*. The 24-h *in vitro* Matrigel invasion assay revealed that miR-145 overexpression decreased glioma cell invasiveness by 63.6% compared to control cells, and the miR-145 downregulation significantly increased invasiveness by 1.53-fold compared to control cells. *In vitro* invasion assay indicated that the invasiveness of U87 glioma cells is correlated to miR-145 levels. ^*^P<0.05 versus control.

We employed the Matrigel invasion assay to test if overexpressing miR-145 affects glioma cell invasiveness. Invasive cells were measured at 24 h after addition of cells into the upper chamber. miR-145 overexpression significantly decreased cell invasion by 63.6% in U87 (Fig. 3). However, the invasiveness of miRzip-145 transfected glioma cells was significantly increased by 1.53-fold compared to control cells. The *in vitro* invasion assay data indicated that the invasiveness of U87 glioma cells is related to miR-145 expression levels, and increase of miR-145 decreases the invasiveness in U87 cells.

### Overexpression of miR-145 inhibits tube formation in cultured mouse brain endothelial cells

**Figure 4:**
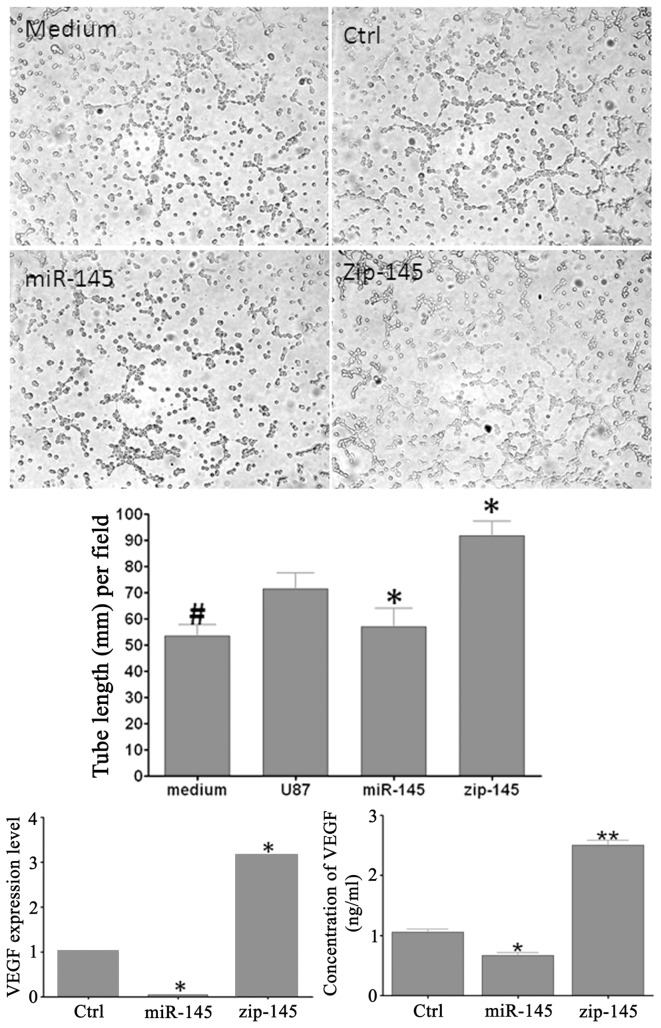
miR-145 overexpression in glioma cells inhibits tube formation in MBECs. Culture medium from U87 miR-145 cells decreased MBECs capillary tube formation by 34.55% compared to control medium. Medium from U87 zip-145 cells promoted capillary tube formation by 1.28-fold compared to control. As compared with medium only, U87 conditioned medium increased the tube formation in MBECs. Overexpression of miR-145 signficantly decreased VEGF expression in U87 miR-145 cells and soluble VEGF release after 24 h. While zip-145 significantly promoted VEGF expression in U87 zip-145 cells and soluble VEGF release. ^*^P<0.05 versus control; ^**^P<0.05 versus control.

To test the effect of miR-145 expression upon glioma-induced angiogenesis, we performed a tube-formation assay of mouse brain endothelial cells (MBECs) *in vitro*. As shown in Fig. 4, culture medium from U87 glioma overexpressing miR-145 significantly reduced capillary tube formation of mouse brain endothelial cells by 34.55% when compared to control U87 media. While down-regulation of miR-145 medium showed a significant 1.28-fold increase in MBECs capillary tube formation compared to control medium. These results suggest that miR-145 in glioma cells promotes angiogenesis.

### Overexpression of miR-145 decreases VEGF in mRNA and soluble protein release levels

Real-time PCR was employed to measure VEGF mRNA expression level in U87 miR-145, U87 zip-145, and control U87 glioma cells (Fig. 4). VEGF expression level was significantly increased (3.16-fold) in U87 zip-145 cells, and was significantly decreased by 95.13% in U87 miR-145 cells compared to control cells. These results indicate that miR-145 regulates VEGF mRNA expression.

VEGF soluble protein release showed the same profile as mRNA levels in all experimental groups. miR-145, zip-145, control U87 cells were cultured under 37°C for 24 h to attached to the bottom of the 6-well plate, respectively. Following complete removal of medium 24 h later, glioma cells were rinsed by PBS and serum-free medium was added for another 24 h. Culture medium was then collected for analysis of soluble VEGF release from glioma cells (Fig. 4). High miR-145 expression decreased to 62.4% the VEGF release to supernatant by U87 miR-145, and low expression of miR-145 increased VEGF release to 2.4-fold by U87 zip-145.

### ADAM17, EGFR, and Erk phosphorylation in miR-145 over-expressing cell lines

**Figure 5:**
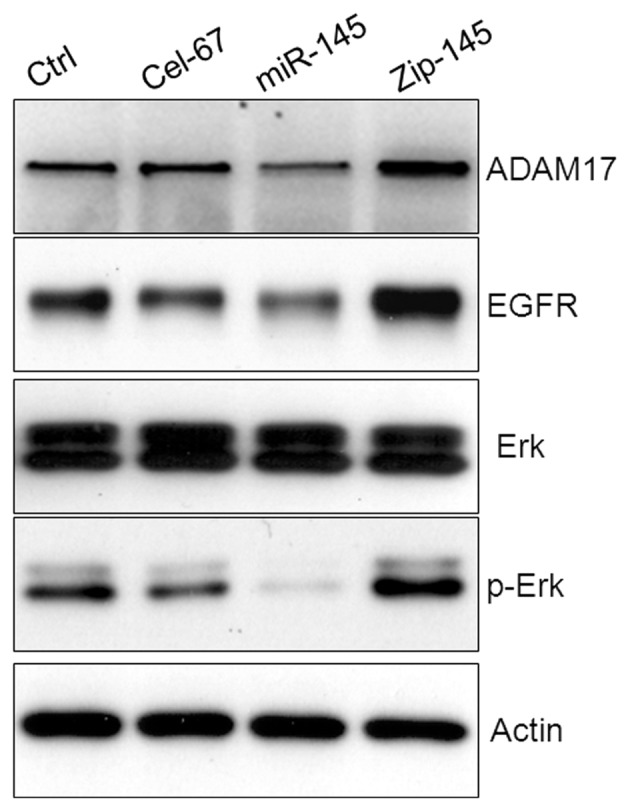
Effect of miR-145 overexpression or downregulation on ADAM17/EGFR/ERK activation in U87 cells. miR-145 was reported to directly target ADAM17 and EGFR. ADAM17 and EGFR expression showed a significant decrease in U87 miR-145 cells and a significant increase in U87 zip-145 cells. Expression of p-Erk showed the same trend as ADAM17/EGFR, but expression of Erk was not significantly affected by miR-145 expression levels.

To test the hypothesis that ADAM17/EGFR/ERK/p-ERK contribute to the miR-145 effect on glioma cells, western blot assay was employed (Fig. 5). The data showed that miR-145 overexpressing U87 cells have lower expression of ADAM17 and EGFR, respectively, in total protein levels, and lower phosphorylation state of Erk as compared with control groups. Furthermore, low expression of miR-145 in glioma cells was correlated to increased ADAM17 and EGFR expression. These data suggest that miR-145 significantly contribute to the pathways known to promote glioma cell malignancy.

### miR-145 inhibits U87 glioma growth in nude mice

**Figure 6:**
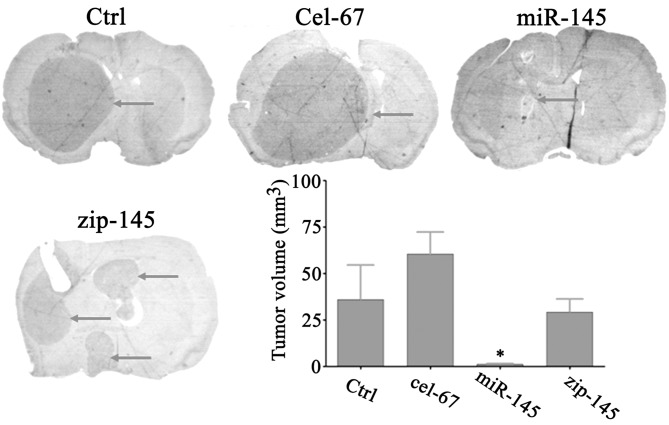
The expression level of miR-145 is related to U87 glioma cell growth in nude mice. U87 miR-145 showed significant decrease in tumor volume (36.25 vs 1.25 mm^3^, ^*^P<0.05) in nude mice compared to control glioma cells. Zip-145 transfected cells showed no significant growth rate as compared with their parent cells. n=6 nude mice/group; ^*^P<0.05 versus control.

MiR145-overexpressing U87 glioma cells were transfected with negative plasmid cel-67, and: i) control; ii) cel-67; iii) miR-145; and iv) zip-145 U87 glioma cells were injected stereotactically (5×10^5^ cells/mouse) at a depth of 3.5 mm, 1.5 mm to the right of midline, and 1.0 mm anterior to the bregma. Animals were sacrificed at 30 days after tumor implantation. Tumor-bearing brain was processed, placed in paraffin, and subsequently seven equally spaced, 6 μm sections, were obtained from each of the 1-mm blocks encompassing the tumor. The sections were stained with H&E for light microscopic examination and analysis. After 30 days, miR-145 transfected U87 cells showed a significant decrease in tumor volume (Fig. 6) in nude mice compared to control cells and parent glioma cells transfected with cel-67. As shown in Fig. 6, zip-145 transfected U87 cells grew to form a solid tumor and showed no significant increase in tumor volume in comparison with control. The nude mice with U87 zip-145 cell implantation died 14 days after implantation. These data indicate that miR-145 significantly contributes to glioma xenograph growth, and suggests overexpression of miR-145 may have therapeutic antitumor effects for glioma.

## Discussion

Glioblastoma is the most common primary brain tumor in adults. Almost all high-grade gliomas will invariably recur due to their extensive invasion into the surrounding brain tissue supported by their high capacity of neovascularization (21). These features cause treatment failure and have a very high mortality rate by conventional therapies. ADAMs are an important family of proteins upregulated in many cancers including glioma (22,23). miR-145 has been reported to target and regulate ADAM17, an important ADAM family member (20,24). In addition, accumulated evidence showed that the non-coding miRNAs have multi-function on gene regulation during tumor progression (25).

In the present study, the U87 human glioblastoma cell line was employed to investigate to the effect of miR-145 on glioma cell proliferation, invasion, and angiogenesis *in vitro*. Overexpression and downregulation of miR-145 were established with transfection of miR-145 and zip-145 plasmids, respectively. Growth curve and BrdU incorporation assays were employed to examine the effect of miR-145 on U87 glioma cell proliferation. Our data demonstrate that miR-145 decreases U87 glioma cell proliferation. Decrease of miR-145 by zip-145 significantly increased tumor cell proliferation. These data suggest that miR-145 plays a key role in U87 cell malignancy. We used Matrigel invasion assays to assess the ability of glioma cells to penetrate the ECM. Our data showed that the invasion potential of U87 cells is decreased by miR-145 overexpression, and increased by miR-145 downregulation.

Neovascularization plays critical roles in the process of embryo development, tissue differentiation and repair, inflammatory diseases, and tumor growth and metastasis (26,27). During the angiogenic process, endothelial cells digest the basement membrane of the vessel, migrate through the membrane while they proliferate in number, form a new vessel lumen and then sprout into a new vessel branch (28). Proliferation and growth of tumor cells consume oxygen in solid tumors. The hypoxia and necrosis resulting from rapid growth triggers the angiogenic switch out of the vascular quiescent state, which subsequently supports tumor progression (29). The initiation of newly formed tumor vessels also known as neovascularization or angiogenesis requires pro-angiogenic growth factors including vascular endothelial growth factor (VEGF) and related molecules. VEGF can be triggered by extracellular signals such as transforming growth factor α (TGF-α) and hypoxia-inducible factor 1-α (HIF-α), and internal genetic change (29,30). ADAM17 and EGFR increase the expression of HIF-α, and HIF-α then activates VEGF and VEGFR to stimulate tumor angiogenesis (22,31). To address the function of miR-145 on glioma angiogenesis, we further tested VEGF expression in U87 cells and angiogenesis *in vitro* with mouse brain endothelial cells cultured in supernatant from U87 cells. Real-time PCR, soluble protein, and tube formation assays demonstrated that high expression of miR-145 leads to a decrease in gene, protein and functional levels of VEGF. VEGF plays an important role in glioma progression. An *in vitro* capillary tube formation assay was employed to determine the effect of miR-145 on angiogenesis. Our data suggest that miR-145 decreased capillary-like tube formation. However, downregulation of miR-145 reverses the capacity of tube formation that was associated with lowered VEGF expression. Therefore, our data suggest downregulation of VEGF expression may be one of mechanisms by which miR-145 inhibits angiogenesis. We found that miR-145 expression was inversely correlated with VEGF mRNA, in addition to VEGF protein. This suggests that the effect of miR-145 upon VEGF may be indirect, rather than via translational inhibition. It is also likely that other factors or pathways are involved in the regulation of neovascularization. Therefore, further investigation of other signaling factors or pathways is warranted.

In our *in vivo* study, the data indicate that miR-145 is a growth inhibitor in U87 human glioma progression. Stable transfection with a plasmid encoding miR-145 leads to inhibition of the malignant phenotype. miR-145 overexpression decreased the rate of tumor growth in U87-miR-145 glioma bearing nude mice as compared with those parent tumor control and negative tumor control. Downregulation of miR-145 promotes tumor invasion and tumor growth. These data confirmed our *in vitro* result that increased miR-145 expression decreases glioma proliferation.

ADAM17 is a primary sheddase for multiple EGFR pro-ligands, such as HB-EGF and TGF-α (13,32). EGFR can be activated by its ligands including EGF, TGF-α, amphiregulin, and betacelluin (14,33). It is the first identified receptor tyro-sine kinase (34). EGFR is amplified and overexpressed in tumors of many tissues (35–37). EGFR and its downstream signaling pathway is a key regulator of cell proliferation and it is frequently deregulated in cancer (38,39). EGFR ligand-binding results in receptor self-dimerization, auto-phosphorylation, and subsequent activation of downstream PI3K/AKT and Ras/MAPK pathways, which are responsible for the malignant phenotype (14,15). Furthermore, we examined the mechanisms by which ADAM17/EGFR/MAPK/ERK pathway contributed to miR-145-induced inhibition on glioma proliferation, invasion, and angiogenesis after transfection of miR-145. High expression of miR-145 resulted in a significant decrease in U87 cell proliferation, invasion and angiogenesis. Coincidentally, miR-145 overexpression deactivated ADAM17/EGFR/ERK *in vitro*, and downregulation of miR-145 increased ADAM17/EGFR/ERK activation. These data further indicate that miR-145 overexpression contributes to reduction of tumor progression through deactivation of the ADAM17/EGFR/ERK pathway.
